# Author Correction: Extreme conditions affect neuronal oscillations of cerebral cortices in humans in the China Space Station and on Earth

**DOI:** 10.1038/s42003-022-04086-1

**Published:** 2023-02-02

**Authors:** Peng Zhang, Juan Yan, Zhongqi Liu, Hongqiang Yu, Rui Zhao, Qianxiang Zhou

**Affiliations:** 1 School of Biological Science and Medical Engineering, Beihang University, Beijing, 100191 China; 2 Beijing Advanced Innovation Center for Biomedical Engineering, Beihang University, Beijing, 100191 China; 3 China CDC Key Laboratory of Radiological Protection and Nuclear Emergency, National Institute for Radiological Protection, Chinese Center for Disease Control and Prevention, Beijing, 100088 China; 4 China Astronaut Research and Training Center, Beijing, 100193 China

**Keywords:** Cortex, Working memory

Correction to: *Communications Biology* 10.1038/s42003-022-04018-z, published online 30 September 2022.

In the original version of this Article, the “EEG acquisition and preprocessing” section within the Methods mistakenly reported extra analyses related to the ICA pipeline parameters, which were not performed in this study:

“In this study, astronauts performed WM tasks involving letter switching in microgravity conditions, where microsaccades are easily caused and capable of producing gamma activity. Spike potentials (SP) have received attention because even involuntary microsaccades (<1°) during attempted fixation generate sizeable SPs, which introduce a broadband artifact in the time-frequency spectrum of the EEG, affecting the low-amplitude beta and gamma bands73 (>30 Hz), in particular. Dimigen74 demonstrated that correction could be strongly improved by training the ICA on optimally filtered data in which SPs were massively overweighted. With optimized procedures, ICA removed virtually all artifacts from both viewing paradigms, including the SP and its associated spectral broadband artifact, with little distortion of neural activity. Thus, we tracked eye movements (RED, SMI, GER) synchronously with EEG signals and employed this advanced ICA algorithm based on SP overweighting. In the first step, four parameters of the ICA pipeline were varied orthogonally: the (1) high-pass and (2) low-pass filter applied to the training data, (3) the proportion of training data containing myogenic saccadic SP, and (4) the threshold for eye tracker based component rejection. In the second step, the eye-tracker was used to objectively quantify the correction quality of each ICA solution, both in terms of under-correction (residual artifacts) and overcorrection (removal of neurogenic activity). Dimigen provided Matlab code in the published paper74”.

This section should instead read as:

In this study, astronauts performed WM tasks involving letter switching in microgravity conditions, where microsaccades are easily caused and capable of producing gamma activity73. Dimigen74 demonstrated that correction could be strongly improved by training the ICA on optimally filtered data in which spike potentials were massively overweighted. Thus, we tracked eye movements (RED, SMI, GER) synchronously with EEG signals and employed an advanced ICA algorithm based on spike potentials overweighting, which was explored by Dimigen. Dimigen provided Matlab code in the published paper74.

This error has now been corrected in the PDF and HTML versions of the Article.

