# Supplementary material for: Extreme conditions affect neuronal oscillations of cerebral cortices in humans in the China Space Station and on Earth
Source: Commun Biol. 2022 Sep 30;5:1041. doi: 10.1038/s42003-022-04018-z (PMC9525319; doi:10.1038/s42003-022-04018-z)
Supplement: Supplementary file 2 — Supplemental Material [file 42003_2022_4018_MOESM2_ESM.pdf]

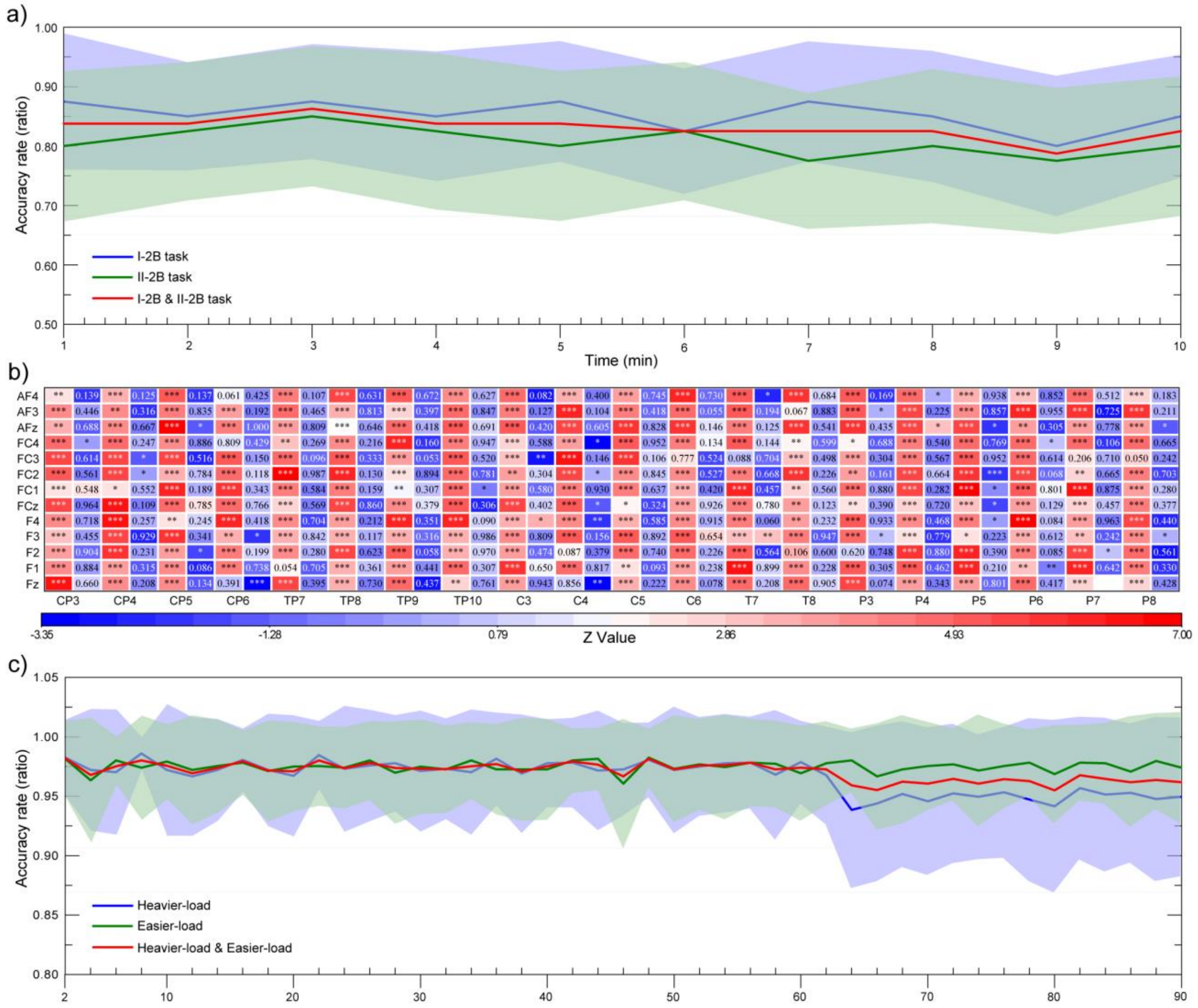

Supplementary Fig. 1: Results of the lengthy mental task. n = 54, **a)** Behavior performance (the mean accuracy rate of the two-back task) changed with the sleep deprivation and recovery time course. I-2B and II-2B tasks represented the two-back tasks before and after the lengthy mental task, respectively. Accuracy rates of I-2B, II-2B task and their average are shown by a blue, green, and red line, respectively. The shading represents standard deviations. **b)** One-tailed FDR-corrected Wilcoxon tests were used to reveal if phases of an electrode in the frontal-central cortex (FCC) significantly modulated amplitudes of another electrode in the temporal-parietal cortex (TPC). ***, ** and * represent $p < 0.0001$, < 0.001 and < 0.05, respectively. Dark blue to dark red indicates Z-values gradually increase. For each electrode marked on the horizontal axis, the first column is the statistical result before the lengthy mental task and the second column is the statistical result after the lengthy mental task. **c)** Behavior performance (the mean accuracy rate of the lengthy mental task) changed with the sleep deprivation and recovery time course. The blue, green, and red lines show the accuracy rates of heavier-load, easier-load conditions, their average. The shading represents standard deviations.

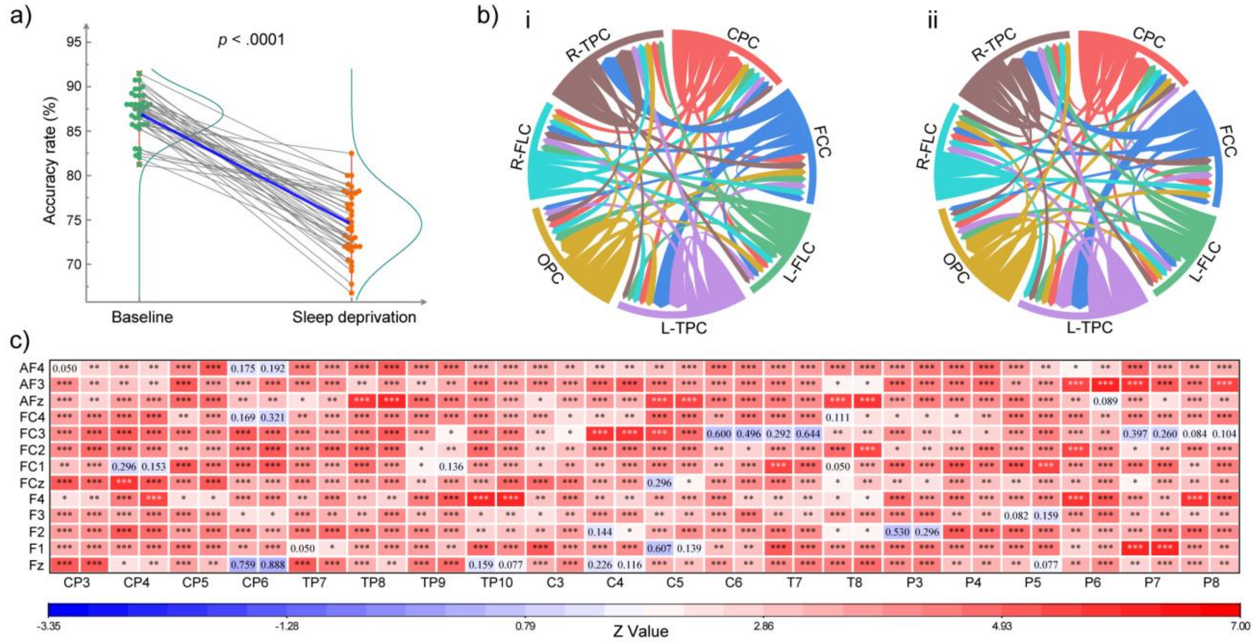

Supplementary Fig. 2: Results of the sleep deprivation. n = 38, **a)** After the sleep deprivation, behavior performance, the mean accuracy rate of the two-back task, was significantly declined indicated by a one-way repeated ANOVA. **b)** Before (**i**) and after (**ii**) sleep deprivation, chord diagrams from the ergodic analysis show electrode clusters in the temporal-parietal cortex (TPC) having a great majority of gamma amplitude modulation by theta phase obtained from electrode clusters in the frontal-central cortex (FCC). The no change in the thickness of the connecting lines indicates that theta-gamma phase-amplitude coupling (PAC) between TPC and FCC was not decoupled after the sleep deprivation. **c)** One-tailed FDR-corrected Wilcoxon tests were used to reveal if phases of an FCC electrode significantly modulated amplitudes of another TPC electrode. ***, ** and * represent $p < 0.0001$, < 0.001 and < 0.05, respectively. Dark blue to dark red indicates *Z*-values gradually increase. For each electrode marked on the horizontal axis, the first column is the statistical result before the sleep deprivation (baseline) and the second column is the statistical result after the sleep deprivation.

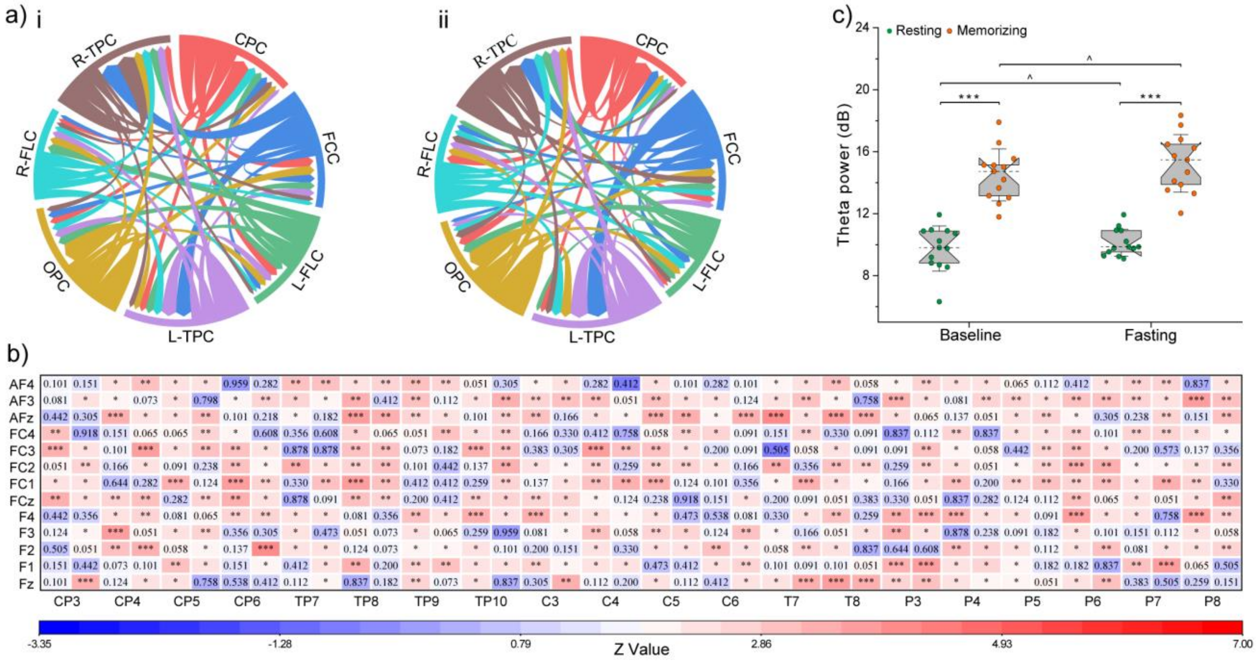

Supplementary Fig. 3: Results of the complete fasting. n = 13, **a)** Before (**i**) and after (**ii**) complete fasting, chord diagrams from the ergodic analysis show electrode clusters in the temporal-parietal cortex (TPC) having a great majority of gamma amplitude modulation by theta phase obtained from electrode clusters in the frontal-central cortex (FCC). The no change in the thickness of the connecting lines indicates that theta-gamma phase-amplitude coupling (PAC) between TPC and FCC was not decoupled after the complete fasting. **b)** One-tailed FDR-corrected Wilcoxon tests were used to reveal if phases of an FCC electrode significantly modulated amplitudes of another TPC electrode. ***, ** and * represent $p <$ 0.0001, < 0.001 and < 0.05, respectively. Dark blue to dark red indicates *Z*-values gradually increase. For each electrode marked on the horizontal axis, the first column is the statistical result before the complete fasting (baseline) and the second column is the statistical result after the complete fasting. **c)** The averaged theta wave activity (TWA) in FCC of the memorizing (orange dots) condition was significantly higher than that of the resting (green dots) condition (*** : $p < 0.0001$). After the complete fasting, TWA in FCC was not significantly different (^ : $p > 0.05$). Data is shown by boxplot. The whisker threads in both ends of the boxplot represent standard deviations, and dashed lines in the middle of the boxplot represent medians.

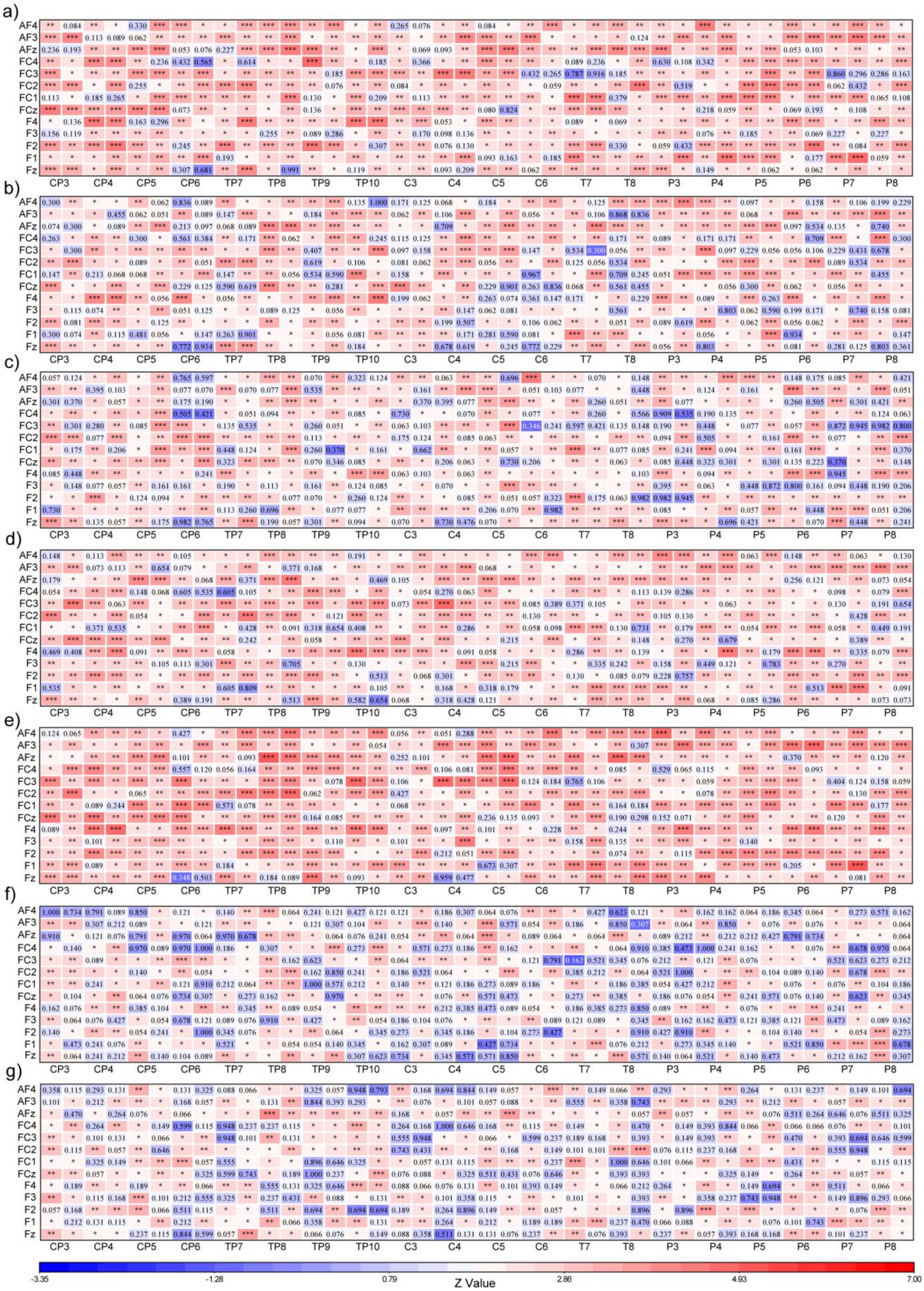

Supplementary Fig. 4: Theta-gamma phase-amplitude coupling (PAC) of the extreme psychological situation. One-tailed FDR-corrected Wilcoxon tests were used to reveal if phases of an electrode in the frontal-central cortex (FCC) significantly modulated amplitudes of another electrode in the temporal-parietal cortex (TPC). ***, ** and * represent $p < 0.0001$, < 0.001 and < 0.05, respectively. Dark blue to dark red indicates Z-values gradually increase. For each electrode marked on the horizontal axis, the first

column is the statistical result of the normal mood (baseline), and the second column is the statistical result of the abnormal mood. The statistical results of excitement (n = 22), anger (n = 15), fear (n = 14), sadness (n = 17), anxiousness (n = 24), hate (n = 10), and depression (n = 11) are shown in figures **a), b), c), d), e), f),** and **g)**, respectively.

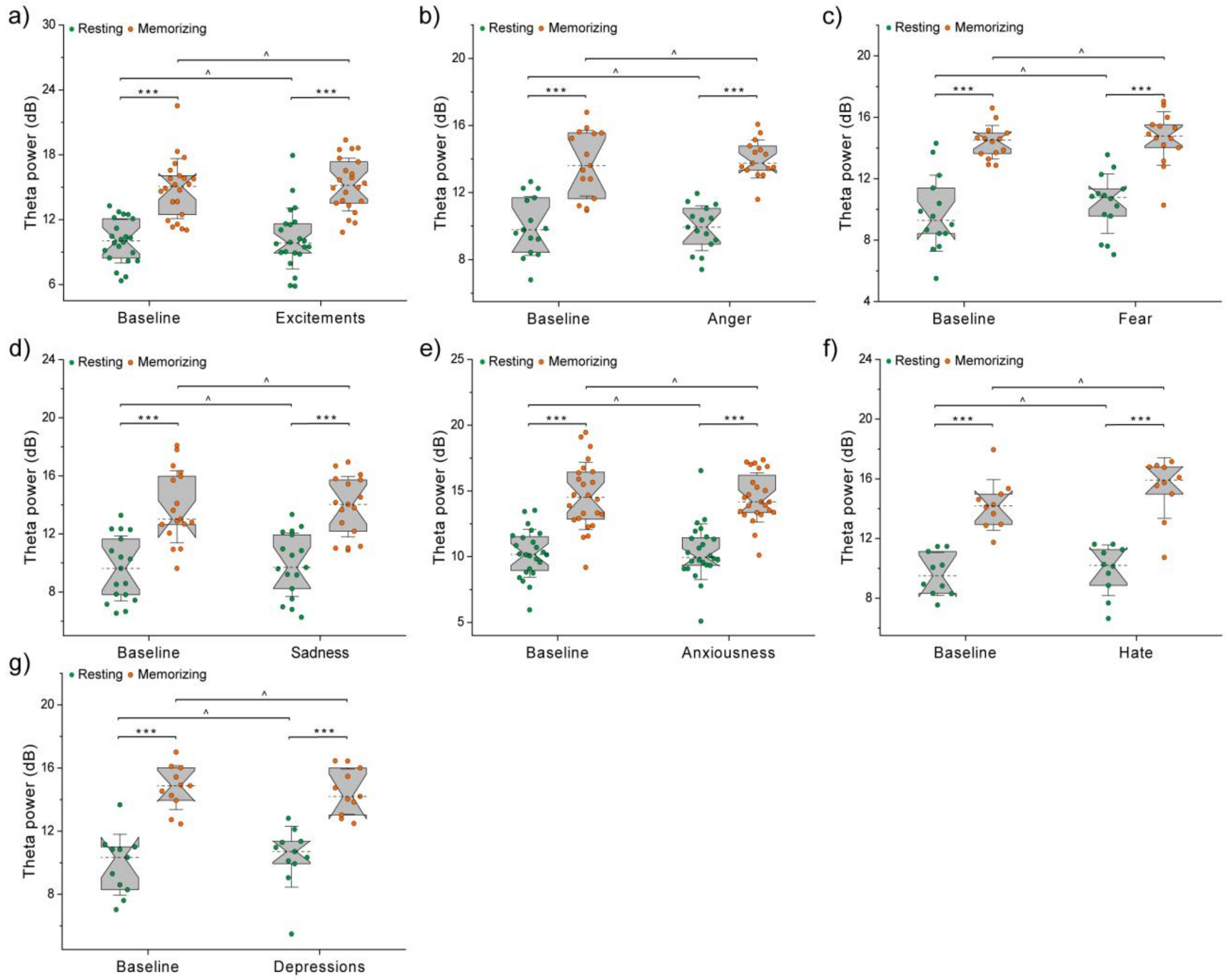

Supplementary Fig. 5: Theta wave activity (TWA) of the extreme psychological situation. For frontal-central cortex (FCC), these pictures show that the averaged theta wave activity (TWA) of memorizing (orange dots) condition was significantly higher than that of the resting (green dots) condition (*** : $p < 0.0001$). TWA in FCC was not significantly different (^ : $p > 0.05$) in the extreme psychological situation. The statistical results of excitement (n = 22), anger (n = 15), fear (n = 14), sadness (n = 17), anxiousness (n = 24), hate (n = 10), and depression (n = 11) are shown in figures **a), b), c), d), e), f),** and **g)**, respectively. Data is shown by boxplot. The whisker threads in both ends of the boxplot represent standard deviations, and dashed lines in the middle of the boxplot represent medians.

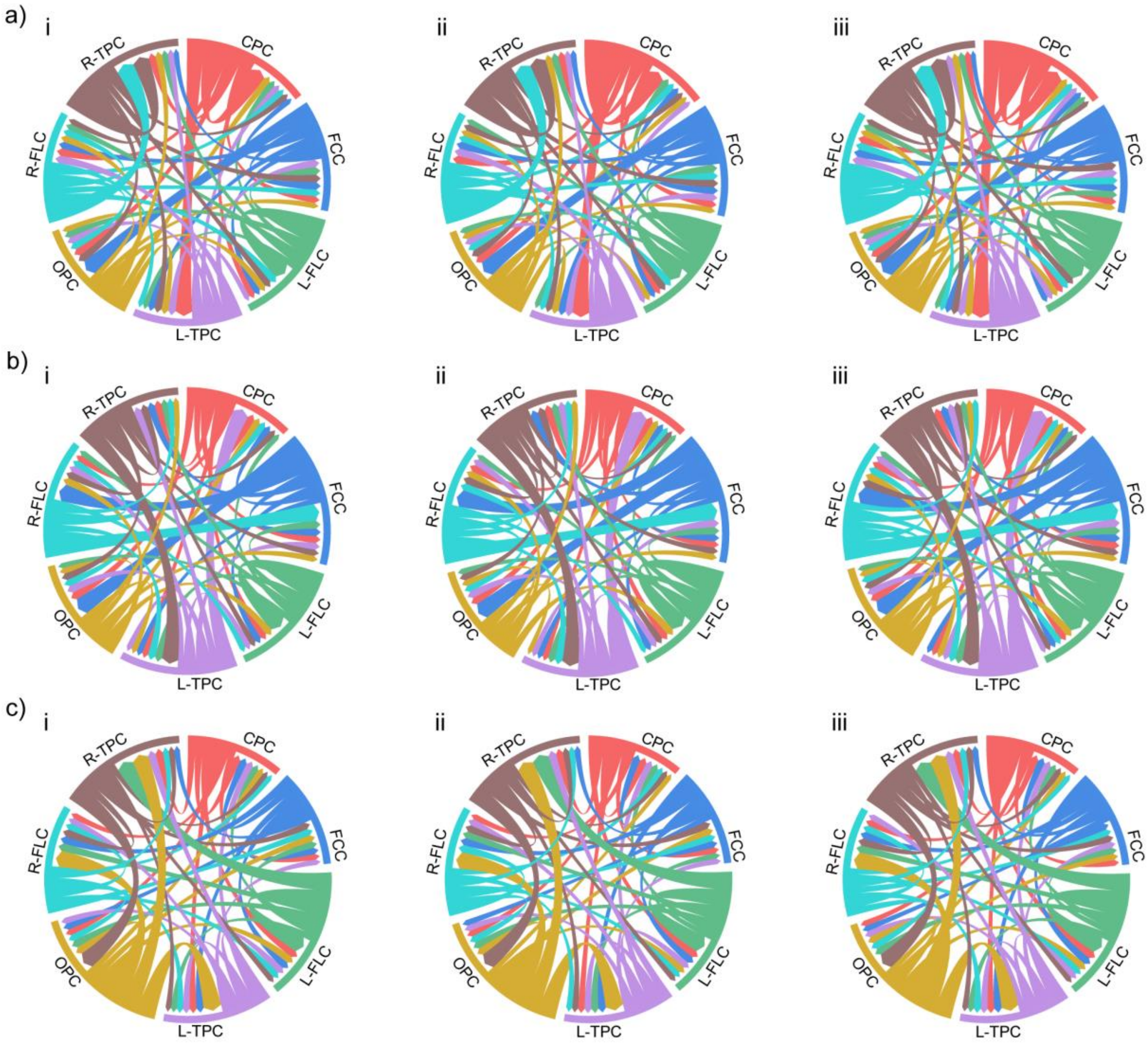

Supplementary Fig. 6: Results of brain rhythm pairs. n = 54, **a), b)** and **c)** show PAC rhythm pairs of θ-β, α-β, and α-γ, respectively. The **i**, **ii**, and **iii** panels represent baseline status, tests after length mental tasks, and after sleep deprivations.

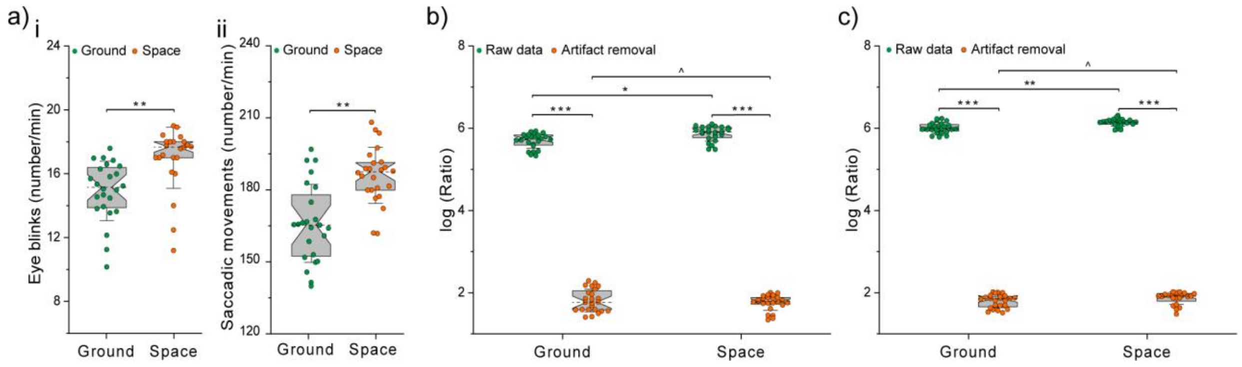

Supplementary Fig. 7: Results of eye tracking and artifact removal. n = 24, **a)** The occur times per minute of spontaneous eye blinks, and saccadic eye movements are shown in panel **i** and **ii**, respectively. Green and orange dots represent data measured on the ground and in space, respectively. **b)** and **c)** show the regional blink-to-baseline power ratios before and after artifact rejection in the frontal-central cortex (FCC) and the temporal-parietal cortex (TPC) surface, respectively. Green and orange dots represent raw and artifact removal data, respectively. ^, *, **, and *** indicate $p > 0.05$, $p < 0.05$, $p < 0.001$, $p < 0.0001$, respectively. Data is shown by boxplot. The whisker threads in both ends of the boxplot represent standard deviations, and dashed lines in the middle of the boxplot represent medians.
