# Supplementary material for: Extreme conditions affect neuronal oscillations of cerebral cortices in humans in the China Space Station and on Earth
Source: Commun Biol. 2022 Sep 30;5:1041. doi: 10.1038/s42003-022-04018-z (PMC9525319; doi:10.1038/s42003-022-04018-z)
Supplement: Supplementary file 3 — Description of Additional Supplementary Files [file 42003_2022_4018_MOESM3_ESM.pdf]

# Description of Additional Supplementary Files

**File name:** Supplementary Data 1

**Description:** The source data behind figure 2 in the paper. Data for each panel were stored in an independent sheet of Excel file.

**File name:** Supplementary Data 2

**Description:** The source data behind figure 3 in the paper. Data for each panel were stored in an independent sheet of Excel file.

**File name:** Supplementary Data 3

**Description:** The source data behind figure 4 in the paper. Data for each panel were stored in an independent sheet of Excel file.

**File name:** Supplementary Data 4

**Description:** The source data behind figure 5 in the paper. Data for each panel were stored in an independent sheet of Excel file.
