# Supplementary material for: Extreme conditions affect neuronal oscillations of cerebral cortices in humans in the China Space Station and on Earth
Source: Commun Biol. 2022 Sep 30;5:1041. doi: 10.1038/s42003-022-04018-z (PMC9525319; doi:10.1038/s42003-022-04018-z)
Supplement: Supplementary file 8 — Reporting Summary [file 42003_2022_4018_MOESM8_ESM.pdf]

nature portfolio

Corresponding author(s): Qianxiang Zhou

Last updated by author(s): Sep 14, 2022

Reporting Summary

Nature Portfolio wishes to improve the reproducibility of the work that we publish. This form provides structure for consistency and transparency in reporting. For further information on Nature Portfolio policies, see our Editorial Policies and the Editorial Policy Checklist.

Statistics

For all statistical analyses, confirm that the following items are present in the figure legend, table legend, main text, or Methods section.

n/a	Confirmed	
☐	☒	The exact sample size (*n*) for each experimental group/condition, given as a discrete number and unit of measurement
☐	☒	A statement on whether measurements were taken from distinct samples or whether the same sample was measured repeatedly
☐	☒	The statistical test(s) used AND whether they are one- or two-sided<br>*Only common tests should be described solely by name; describe more complex techniques in the Methods section.*
☐	☒	A description of all covariates tested
☐	☒	A description of any assumptions or corrections, such as tests of normality and adjustment for multiple comparisons
☐	☒	A full description of the statistical parameters including central tendency (e.g. means) or other basic estimates (e.g. regression coefficient) AND variation (e.g. standard deviation) or associated estimates of uncertainty (e.g. confidence intervals)
☐	☒	For null hypothesis testing, the test statistic (e.g. *F*, *t*, *r*) with confidence intervals, effect sizes, degrees of freedom and *P* value noted<br>*Give P values as exact values whenever suitable.*
☒	☐	For Bayesian analysis, information on the choice of priors and Markov chain Monte Carlo settings
☒	☐	For hierarchical and complex designs, identification of the appropriate level for tests and full reporting of outcomes
☐	☒	Estimates of effect sizes (e.g. Cohen's *d*, Pearson's *r*), indicating how they were calculated

Our web collection on statistics for biologists contains articles on many of the points above.

Software and code

Policy information about availability of computer code

Data collection	EEG signal were acquired with a 64-channel amplifier system (BrainAmp, Brain Products, GER) and recorded using the manufacturer's software. Data of eye movements were tracked with the eye tracker(RED, SMI, GER).
Data analysis	Data analyses and codes were completed in MATLAB (v.2019b, MathWorks, USA) involving EEGLAB v.2019 with the current source density (CSD) toolbox.

For manuscripts utilizing custom algorithms or software that are central to the research but not yet described in published literature, software must be made available to editors and reviewers. We strongly encourage code deposition in a community repository (e.g. GitHub). See the Nature Portfolio guidelines for submitting code & software for further information.

Data

Policy information about availability of data

All manuscripts must include a data availability statement. This statement should provide the following information, where applicable:

- Accession codes, unique identifiers, or web links for publicly available datasets
- A description of any restrictions on data availability
- For clinical datasets or third party data, please ensure that the statement adheres to our policy

The data and datasets generated during the current study are available from the corresponding author on reasonable request.

Field-specific reporting

Please select the one below that is the best fit for your research. If you are not sure, read the appropriate sections before making your selection.

☐ Life sciences ☒ Behavioural & social sciences ☐ Ecological, evolutionary & environmental sciences

For a reference copy of the document with all sections, see nature.com/documents/nr-reporting-summary-flat.pdf

Behavioural & social sciences study design

All studies must disclose on these points even when the disclosure is negative.

Study description	Quantitative experimental
Research sample	Experiment 1: 60 participants were recruited and 54 participants were retained for statistical analysis (33 males, 21 females, age: 29.6 ± 7.1 years, age range: 21-49 years);<br>Experiment 2: 50 participants were recruited and 38 participants were retained for statistical analysis (24 males, 14 females, age 28.0 ± 3.7 years, age range: 24-36 years);<br>Experiment 3: 25 participants were recruited and 13 participants were retained for statistical analysis (7 males, 6 females, age 34.1 ± 7.7 years, age range: 27-51 years);<br>Experiment 4: 9 astronauts who participated in recent Shenzhou missions of China space station were recruited (7 males, 2 female, age: 47.7 ± 6.3 years, age range: 41-57 years).
Sampling strategy	The sample size of the current study satisfies the small sample theory proposed by William Sealy Gosset , and this sample size is larger than that of other peer articles
Data collection	EEG signals were synchronously recorded from healthy volunteers when they were performing the working memory tasks. EEG data were acquired with a 64-channel amplifier system (BrainAmp, Brain Products, GER). Instructions were given to each participant before the formal task , and each participant was allowed to practice in tutorial mode until he or she became familiar with the task During the rest time, subjects were kept awake the under supervision of researchers.
Timing	Experiment 1: January 10 to May 30, 2021;<br>Experiment 2: June 1 to December25, 2020;<br>Experiment 3: June 11 to September 12, 2021;<br>Experiment 4: April 17, 2021 to August 15, 2022.
Data exclusions	Experiment 1: Four subjects were excluded from this analysis due to extreme scales assessed by the Profile of Mood State, and two subjects were also excluded from all analyses because they had too many blinks and horizontal eye movements;<br>Experiment 2: Ten subjects were excluded from this analysis due to extreme scales assessed by the Profile of Mood State, and two subjects were also excluded from all analyses because they had too many blinks and horizontal eye movements;<br>Experiment 3: Eight subjects were excluded from this analysis due to voluntary demission and four subjects with abnormal psychological situations were excluded from this analysis at the end of the complete fasting period;<br>Experiment 4: Spaceflight data of a female astronaut with long hair were excluded from inflight analysis due to high noise signals , but her data with low noise signals before spaceflight were retained.
Non-participation	Eight volunteers dropped out in experiment 3 and no volunteers dropped out in other experiments.
Randomization	All the participants were right -handed and free of any mental or somatic disorder or medication use. Participants were not affected by neurological or psychiatric disorders and had normal or corrected-to-normal vision. The participants were recruited from the community or a university and required a high school degree or above for inclusion in the study. In addition, all the participants maintained a regular sleep-wake schedule before the study, documented by sleep wristwatches (Huawei Watch D, CHN). The Profile of Mood State was used to assess the participants' current mood states just before and after the experiment. These participants with abnormal mood or poor sleep were invited to participate in these experiments on another day.

Reporting for specific materials, systems and methods

We require information from authors about some types of materials, experimental systems and methods used in many studies. Here, indicate whether each material, system or method listed is relevant to your study. If you are not sure if a list item applies to your research, read the appropriate section before selecting a response.

Materials & experimental systems

n/a	Involved in the study
☒	☐ Antibodies
☒	☐ Eukaryotic cell lines
☒	☐ Palaeontology and archaeology
☒	☐ Animals and other organisms
☐	☒ Human research participants
☒	☐ Clinical data
☒	☐ Dual use research of concern

Methods

n/a	Involved in the study
☒	☐ ChIP-seq
☒	☐ Flow cytometry
☒	☐ MRI-based neuroimaging

Human research participants

Policy information about studies involving human research participants

Population characteristics	See above
Recruitment	The participants were recruited from the community or a university and required a high school degree or above for inclusion in the study. Individuals were also compensated for participation . All the participants were right-handed and free of any mental or somatic disorder or medication use. Participants were not affected by neurological or psychiatric disorders and had normal or corrected-to-normal vision . All the participants signed written informed consent before the experiment.
Ethics oversight	The experiments were approved by the local Ethics Committee at the Schoo l of Biol ogical Science and Medical Engineering, Beihang University

Note that full information on the approval of the study protocol must also be provided in the manuscript.
